# Dimension reduction and outlier detection of 3-D shapes derived from multi-organ CT images

**DOI:** 10.1186/s12911-024-02457-8

**Published:** 2024-02-14

**Authors:** Michael Selle, Magdalena Kircher, Cornelia Schwennen, Christian Visscher, Klaus Jung

**Affiliations:** 1 Institute of Animal Genomics, University of Veterinary Medicine Hannover, Hannover, Germany; 2 Institute for Animal Nutrition, University of Veterinary Medicine Hannover, Hannover, Germany

**Keywords:** CT scans, Outlier detection, Dimension reduction, Multiple co-inertia analysis, Bagplots

## Abstract

**Background:**

Unsupervised clustering and outlier detection are important in medical research to understand the distributional composition of a collective of patients. A number of clustering methods exist, also for high-dimensional data after dimension reduction. Clustering and outlier detection may, however, become less robust or contradictory if multiple high-dimensional data sets per patient exist. Such a scenario is given when the focus is on 3-D data of multiple organs per patient, and a high-dimensional feature matrix per organ is extracted.

**Methods:**

We use principal component analysis (PCA), t-distributed stochastic neighbor embedding (*t*-SNE) and multiple co-inertia analysis (MCIA) combined with bagplots to study the distribution of multi-organ 3-D data taken by computed tomography scans. After point-set registration of multiple organs from two public data sets, multiple hundred shape features are extracted per organ. While PCA and *t*-SNE can only be applied to each organ individually, MCIA can project the data of all organs into the same low-dimensional space.

**Results:**

MCIA is the only approach, here, with which data of all organs can be projected into the same low-dimensional space. We studied how frequently (i.e., by how many organs) a patient was classified to belong to the inner or outer 50% of the population, or as an outlier. Outliers could only be detected with MCIA and PCA. MCIA and *t*-SNE were more robust in judging the distributional location of a patient in contrast to PCA.

**Conclusions:**

MCIA is more appropriate and robust in judging the distributional location of a patient in the case of multiple high-dimensional data sets per patient. It is still recommendable to apply PCA or *t*-SNE in parallel to MCIA to study the location of individual organs.

**Supplementary Information:**

The online version contains supplementary material available at 10.1186/s12911-024-02457-8.

## Introduction

Several techniques such as computed tomography (CT) or 3-D cameras are widely used in medicine, biology and agricultural sciences to digitalize 3-dimensional organs, entire bodies or other shapes, resulting in stacks of 2-D images, depth images or 3-D point clouds [1–3]. Representations of organs in 3-D have been employed, for example, to visualize and characterize the stage of liver fibrosis [4] or sexual dimorphism in skeletal anatomy [5]. In livestock animals, a partial 3-D representation of the body has been utilized to derive properties such as body weight and body condition scores [6–8]. In order to describe and distinguish the shapes of the digitalized objects, a large number *d* of features can be extracted from the 3-D data and stored in a feature matrix [9]. Depending on the number *n* of digitalized objects, this feature matrix can have a high-dimensional character, explicitly when $d>n$. Dimension reduction, for example by means of principal component analysis (PCA), can then be used to visualize the distribution of the *n* objects, e.g. for the purpose of identifying directions of variation or clusters and outliers. In particular, in the case of patients, the visualization can help to specify a ‘normal’ or reference population and individuals that deviate from this group. In medicine, the quantitative description of reference populations is helpful to classify patients and thus for clinical decision-making [10, 11]. In a low-dimensional space, a normal population can for example be defined as individuals within the range of specified multivariate quantiles [12]. Both in humans and animals, a reference population can then be used to deduce ‘reference intervals’ for various clinically relevant features, for instance hematologic and biochemical analytes from blood samples. These intervals may vary among different sexes, ages, genetic backgrounds, etc. [13, 14].

In scenarios where different entities of the same individual or object are digitalized, each entity can have a different set of features extracted from its respective 3-D shape. A typical example of such entities are multiple organs from the same CT scan of one patient. Since each entity can have its own feature space, separate visualizations and eventually different clusterings can occur for the whole group of individuals after dimension reduction. While most methods for dimension reduction, such as PCA or t-distributed stochastic neighbor embedding (*t*-SNE), project the high-dimensional data of each type of entity into a separate lower-dimensional space, multiple co-inertia analysis (MCIA) allows to project all data matrices into the same space. Consequently, not only the relationship between individuals but also the relationship between the different entities can be studied in the 2-dimensional visualization. CIA has originally been applied to ecological data to investigate species-environment relationships, determining the covariances of two datasets [15], but has also proven valuable as a means to visualize relationships in multi-omics data [16].

Not only the definition of normal or reference populations but also the detection of abnormal or outlying individuals is often desired in the clinical judgement of individuals as well as in exploratory data analysis. For the detection of outliers in the 2- and 3-dimensional representation of data, the bag- and gemplot have been presented as extensions to the 1-dimensional boxplot method [17, 18]. One such example of application is the outlier detection in “omics” data after dimension reduction by PCA [18]. Yet, so far, bagplots for outlier detection have not been combined with MCIA.

Hence, in this manuscript we demonstrate the combination of bagplots with MCIA in direct comparison to two other dimension reduction techniques, PCA and *t*-SNE, on feature matrices derived from multiple pre-segmented organs of human CT scans. In this regard, we further present both the detection of entire individuals and single organs as outliers. This approach may help with early detection of anomalies in the geometry of organs which can be used, for example, as a quality control for segmentation algorithms or before a more sophisticated model is trained on the data. In biological contexts, with sufficent clinical information, this method can serve as an early indicator that a patient might not fit into a designated population. Finally, to facilitate the interpretation of biological and technical outliers, we propose the parallel use of different dimension reduction techniques before outlier detection with bagplots, as the detection of outliers on the level of individuals appears more consistent with MCIA while PCA delivers more diverse results on the level of single organs.

## Methods

In this section, the datasets used for illustration of the approach, as well as the different methods for data processing, point-set registration, extraction of local and global features, the different approaches for dimension reduction and exploratory analysis are being described. Analyses were done using the programming environments R [19] and Python [20].

### Datasets

For this study, two publicly available datasets of CT scans were chosen. The two datasets, CT-ORG [21] and AbdomenCT-1k [22], contain several pre-segmented organs from human whole-body and abdominal scans under various imaging conditions. The CT-ORG dataset was retrieved from The Cancer Imaging Archive [23, 24], the AbdomenCT-1k dataset was retrieved from the official GitHub repository [22]. For each CT scan, a pair of files with a voxel data structure stored in the NIfTI-1 format is available, including either density values in Hounsfield units [25] or the encoding of the organs from segmentation. Based on the annotations, sublayers representing the following organ structures were retrieved: liver, kidneys and lungs from the CT-ORG dataset as well as liver, kidneys, pancreas and spleen from the AbdomenCT-1k dataset. From these datasets, 41 and 50 CT scans that display the entirety of the aforementioned organs were selected for subsequent analyses, respectively.

### Data processing

The data processing was leaning on the work of Pellicer-Valero and colleagues [1]. First, for all CT scans each layer was converted into a binary coded 2-D image separating the organs from the background. To clean up smaller artifacts and to remove inner structures (e.g. hepatic ducts from the liver or bronchi from the lungs), the following morphological operations from the ‘EBImage’ R-package [26] were performed in successive order: opening and closing (applying a 5x5 box kernel each), then filling of enclosed holes. A 3-D mesh representation of the outmost surface was generated for each organ applying the marching cubes algorithm from the ‘rmarchingcubes’ R-package [27]. Next, the orientation of the organs was visually assessed and, where necessary, the surface mesh objects flipped vertically to ensure the same viewpoint for each set of organs. The lungs (excluding the trachea) and the kidneys were divided into left and right pieces. Thereafter, for each organ the largest isosurface was extracted, utilizing the ‘Rvcg’ R-package [28], to further improve the mesh quality.

### Point-set registration

**Fig. 1:**
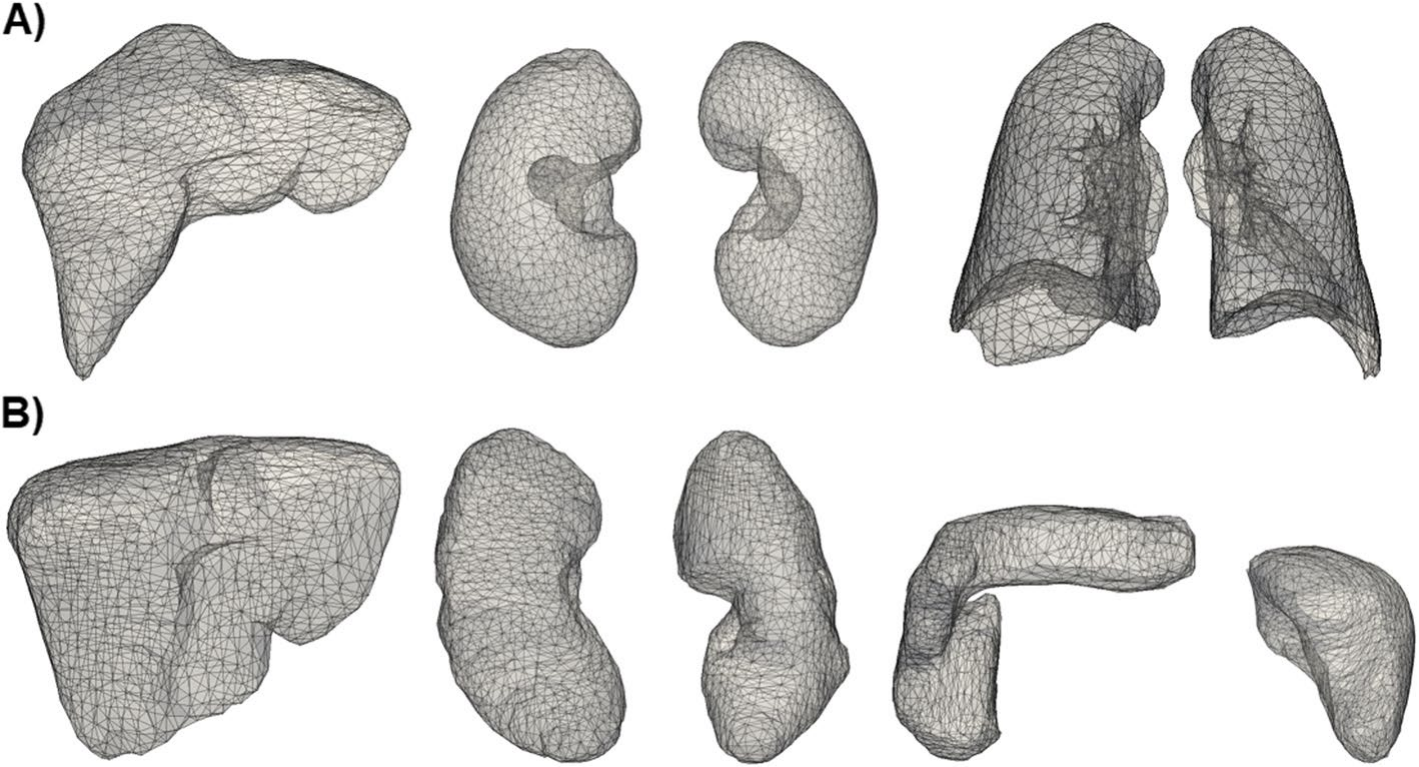
Template Mesh Objects for Point-Set Registration. **A** left to right: liver, kidneys and lungs (CT-ORG dataset); **B** left to right: liver, kidneys, pancreas and spleen (AbdomenCT-1k dataset)

To enhance processing performance, the mesh objects were reduced to approximately 1000 vertices each using the ‘PyVista’ Python-library [29]. Then, surfaces were smoothened via taubin smoothing [29]. At last, all mesh objects were mean-centered. A template mesh object with a volume close to the median volume was selected for each dataset and organ (Fig. 1). Subsequently, the remaining mesh objects were aligned to the respective templates by a two-step coherent point drift algorithm [30]. They were aligned first affinely, then non-rigidly, using the ‘probreg’ Python-library [31]. Finally, spatial correspondence between pairs of points was established applying the Hungarian algorithm from the ‘SciPy’ Python-library [32]. In our scenario, the idea of the Hungarian algorithm is to minimize a cost function that reflects the sum of costs for assigning pairwise the vertices from the two mesh objects. It starts with an $(n \times m)$- adjacency matrix ${\textbf {C}}$, with rows representing the *n* vertices of the first object and columns representing the *m* vertices of the second object. For each pair of row and column, the entry of ${\textbf {C}}$ provides the cost for assigning the two related vertices. In our case, the cost is the Euclidean distance between two vertices from the two mesh objects. Thus, a large distance represents a high cost. The algorithm proceeds then as follows. First the minimum per column is identified and the related entry $c_{i,j}$ is set to zero ($i=1,...,n$; $j=1,...,m$). Next, the same procedure is run to set the minima of rows to zero. Finally, in order to minimize the cost function $\sum \nolimits _{i;j}c_{i;j}$, the aim is to find an assignment by choosing exactly one zero per row and column. If such an assignment is not uniquely available, additional steps are required to minimize the cost function. For further details, we refer to [33].

### Feature extraction

A feature matrix with the hereafter mentioned shape descriptors was generated using the ‘PyVista’ library [29]. After ensuring that the vertex normals for each mesh object face inwards, an array of the pointwise mean curvature was derived from each registered mesh object. Then, sampled ratios of Euclidean distances to geodesic distances between two landmarks were calculated. For that, 500 combinations of two landmarks were randomly selected for each set of registered mesh objects. For reproducibility, a seed was set to obtain features conforming with the same landmarks for each organ. At last, surface area and volume were directly derived from each mesh object and their ratio stored in the feature matrix.

### Methods for dimension reduction

The feature matrices were standardized (i.e. mean-centered and with unit variance) prior to subsequent analyses. For both datasets, the organs from each feature matrix were projected into 2-D space either individually by PCA [34] or t-SNE [35] or for all organs together by MCIA [16], using the R-packages ‘stats’, ‘tsne’ and ‘omicade4’, respectively.

Dimension reduction techniques may be used to project high-dimensional data into lower dimensions while aiming to retain most of the distributional structure of the data [35]. PCA is one of the most well-known dimension reduction techniques with applications in various fields. PCA aims to project observations from one dataset along directions with maximum variation. These directions, obtained from eigenvalue decomposition, called principal components, are linear combinations of the original variables. By separating dissimilar observations, clusters or outliers may be revealed [34, 36].

The *t*-SNE method is a nonlinear dimension reduction technique that focuses on keeping very similar observations close together, i.e. aiming to preserve the local structure within one dataset. *t*-SNE first assesses the probability distribution of pairs of observations in the high-dimensional space and tries to find a similar distribution in the low-dimensional space by minimizing the Kullback-Leibler divergence between the two distributions [35].

Multiple Co-Inertia Analysis (MCIA), as a generalization of CIA, is used to find correlated structure between two or more datasets with matched observations, whereas the variables among all datasets may differ. In this work, organ data from the same individuals but with different shape descriptors for each organ were used. Each dataset is then being projected into the same low-dimensional space [37]. In addition, a common center point is produced, also termed the ‘synthetic center’, which links the same observation from all datasets together. The tighter the linkage, the higher the correlation among different datasets [16].

With respect to our scenario, we briefly summarize the mathematical concept of CIA and MCIA as described in references [16, 38]. While MCIA allows to analyse more than two data sets, CIA is restricted to two data-matrices with *n* matched samples (columns). Let ${\textbf {X}}$ be a mean-centered ($d_1 \times n$)-matrix and ${\textbf {Y}}$ a mean-centered ($d_2 \times n$)-matrix, and both matrices provide point clouds in the high-dimensional space. The term inertia describes the variability for each of these point clouds. For both matrices, we introduce the Euclidean metric ${\textbf {Q}}$ ($d_1 \times d_1$) and ${\textbf {R}}$ ($d_2 \times d_2$), respectively, as well as a weight ($n \times n$)-matrix ${\textbf {W}}=diag(w_1,...,w_n)$. The inertia for ${\textbf {X}}$ and ${\textbf {Y}}$ is then given by

1 $$\begin{aligned} I_{\textbf {X}}=\sum \limits _{i=1}^nw_i||X_i||_Q^2=trace(\mathbf {XQX'W}) \end{aligned}$$

and

2 $$\begin{aligned} I_{\textbf {Y}}=\sum \limits _{i=1}^nw_i||Y_i||_R^2=trace(\mathbf {YRY'W}). \end{aligned}$$

If each individual gets the same weight $w_i=1/n$, the inertia is a sum of variances. The co-inertia describes the geometric correlation between two point clouds and is given by

3 $$\begin{aligned} C(I_{\textbf{X}},I_{\textbf{Y}})&= \sum \limits _{k=1}^{d_1}\sum \limits _{j=1}^{d_2}(\textbf{u}_k'\mathbf {QX'WYR}\textbf{v}_j)^2\nonumber \\&= trace(\mathbf {XQX'WYRY'W}), \end{aligned}$$

where $\textbf{u}_k$ and $\textbf{v}_j$ are sets of $d_1$ and $d_2$ orthogonal vectors that arise when decomposing inertias in formulae (1) and (2). The CIA aims to find first vectors $\textbf{u}_k$ and $\textbf{v}_j$ such that the covariance between the projection of $\textbf{X}$ on $\textbf{u}_k$ and the projection of $\textbf{Y}$ on $\textbf{v}_j$ maximizes the squared covariance $Cov^2(\textbf{XQu}_k, \textbf{YRv}_j)$.

MCIA generalizes this concept to scenarios with $S\ge 2$ data sets $\textbf{X}_s$ ($s=1,...,S$). Then, the sum of squared covariances of each data set and synthetic axes *h* is to be maximized:

4 $$\begin{aligned} \sum \limits _{s=1}^SCov^2(\textbf{X}_s \textbf{Q}_s \textbf{u}_s, h). \end{aligned}$$

### Bagplots for determination of location and outlier detection

After dimension reduction, bagplots were used to specify the overall location of each individual organ with respect to the distribution of all organs as well as for outlier detection. Specifically, each individual organ was assigned to one of the following three regions of the whole distribution as typically specified by a bagplot: (1) inside the inner polygon, called the ‘bag’, (2) inside the outermost polygon, the ‘fence’ or (3) outside the outermost polygon, declared as ‘outlier’ region. As a bagplot is the 2-D extension of the boxplot, the bag includes 50% of all observations, comparable to the interquartile range of a boxplot [17]. Then, for each individual the number of organs that attributed to the majority of one bagplot region were counted, in order to study how robust the location of an individual is judged with respect to the three regions. Thus, it can be assessed whether an entire individual or just a single organ belongs to the bulk of a population or can be flagged as an outlier. If many organs of an individual are located at the same bagplot region, it could be concluded that the entire patient belongs to this region. The distribution within the three methods was compared applying the Kruskal-Wallis test followed by pairwise Mann-Whitney-U tests.

## Results

In this section, the dimension reduction and projection of multiple feature matrices into the 2-D space altogether via MCIA as well as separately via PCA and *t*-SNE are shown. The analysis via MCIA is elucidated in more detail. Furthermore, the location robustness and outlier detection via bagplots are depicted.

### Multiple co-inertia analysis

**Fig. 2:**
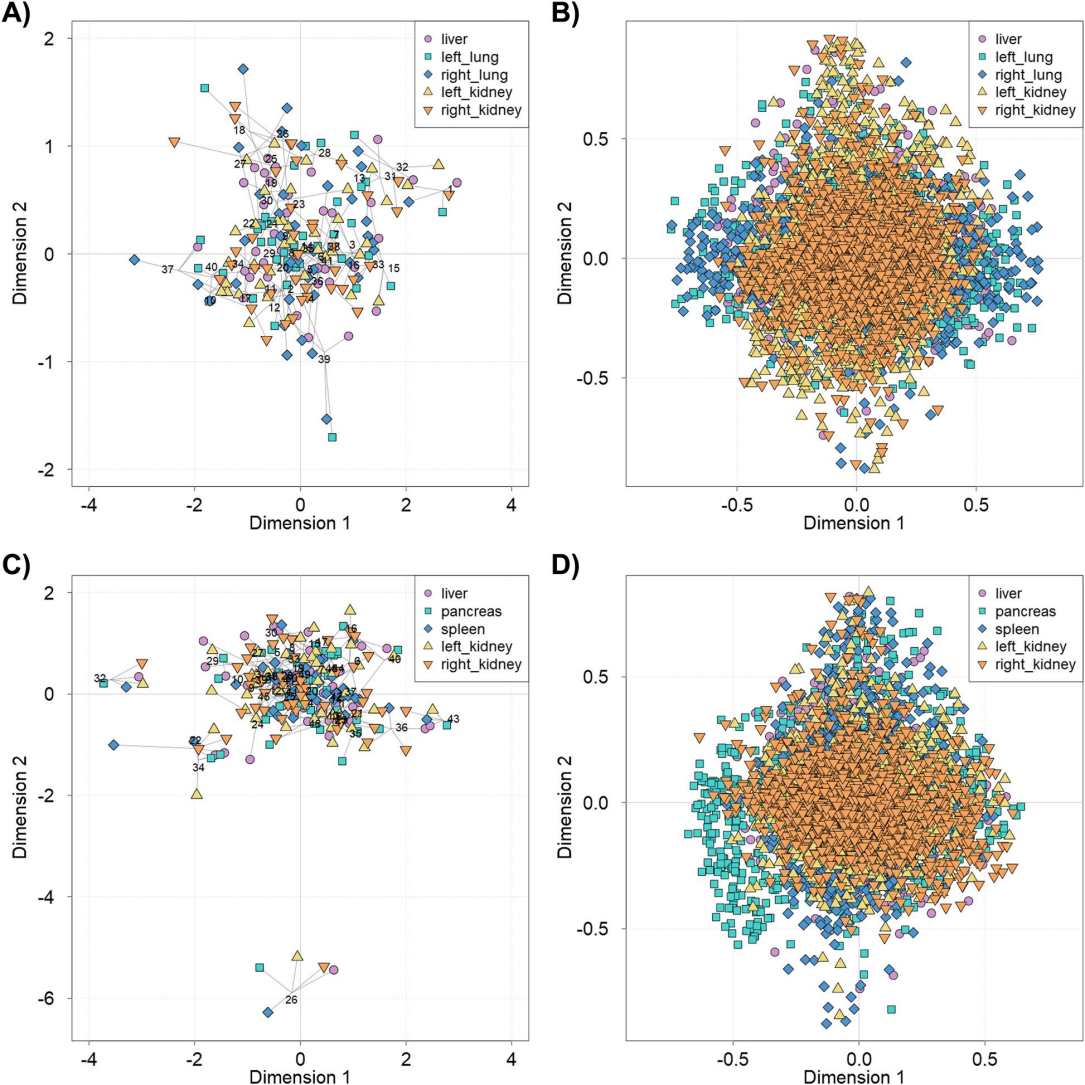
Multiple Co-Inertia Analysis (top: CT-ORG dataset, bottom: AbdomenCT-1k dataset). **A**, **C** Sample space derived from MCIA. Individuals are projected by the geometry of their selected organs into the same 2-D space. Different point shapes illustrate the organ features a sample point is based on. The liver, left lung, right lung, left kidney and right kidney (**A**) as well as liver, pancreas, spleen, left kidney and right kidney (**C**) originating from the same individual are connected by lines that meet at a common center point. The shorter the lines, the higher the correlation of samples. Each individual is labeled by a number. **B**, **D** Variable space projecting each feature from all feature matrices into the same 2-dimensional space. The further away a feature is projected from the point of origin in the same direction as a sample, the higher the value of that feature in the respective sample

For both datasets, CT-ORG and AbdomenCT-1k, the feature matrices with shape descriptors for each organ were projected into the same 2-D space via MCIA (Fig. 2A, C). The number of features per organ amounts to approx. 1,500 features. In the MCIA plot, organs from the same individual are connected by lines to a common center point. While most individuals group closely together, few can be observed that separate more clearly from the others. For example, in the AbdomenCT-1k dataset (Fig. 2C) individuals no. 22, 26, 32, and 34 were projected further apart from most other individuals.

The variable space (Fig. 2B, D) illustrates the contribution of each feature to the lower-dimensional projection of the respective individuals. A variable positioned in the same direction as a sample indicates an elevated feature value in that sample. In turn, a feature facing in the opposite direction of a sample indicates a decreased feature value in that particular sample. The further away the feature is projected from the point of origin, the higher the association on that axis. As for the AbdomenCT-1k dataset, for example, the individual no. 26 was separated more clearly from the population. A closer look at features from the pancreas with $Dimension 2 < -0.5$ in the variable space revealed that some ratios of Euclidean to geodesic distances from proximal to distal ends attributed considerably to the variance. Regarding the geometry of the pancreas, it can be seen that the respective shape is less curved compared to the template (Suppl. Fig. 1), which is located close to the center, and most other shapes. However, the variance within the shape of other organs was often explained by the mean curvature of few vertices.

**Fig. 3:**
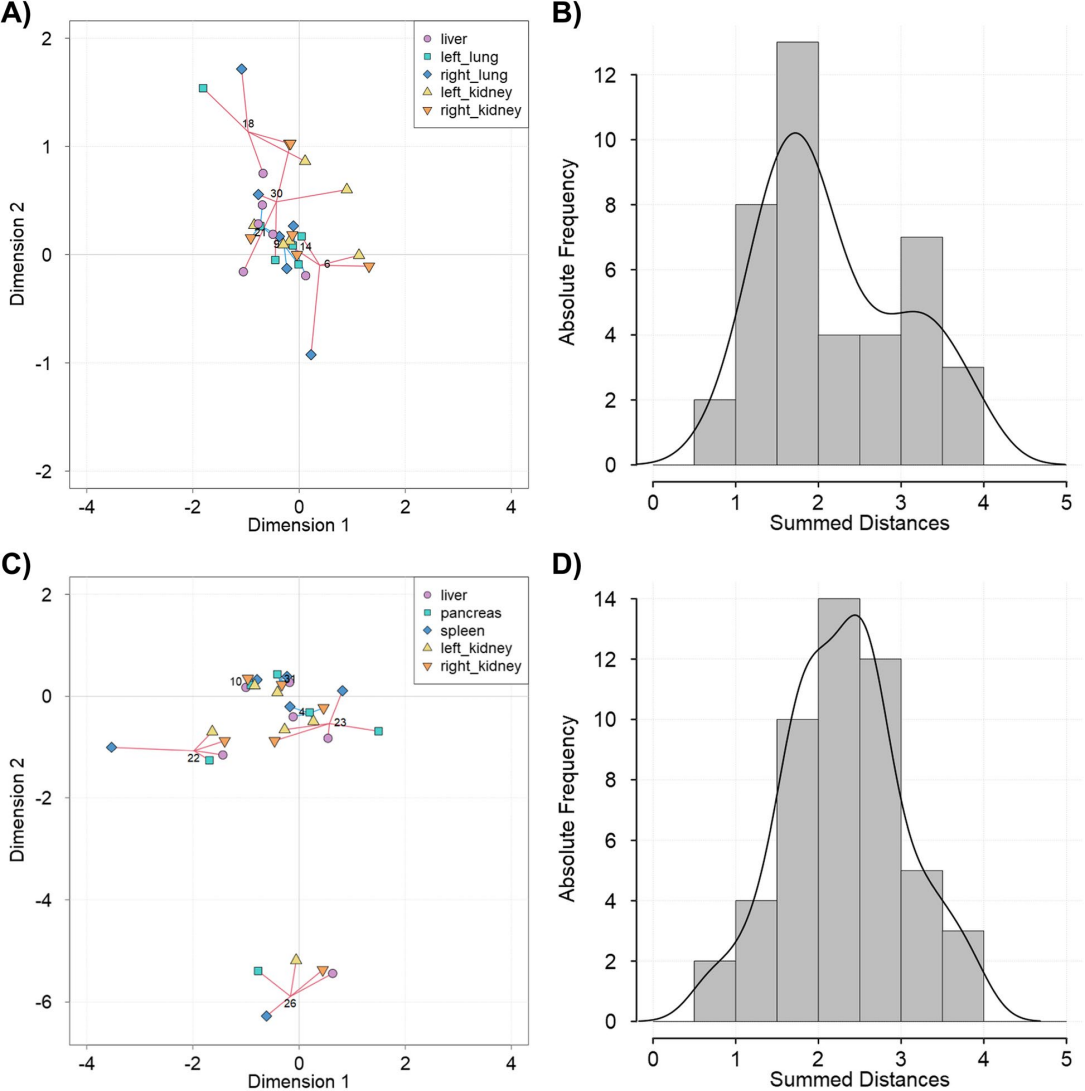
Multi-organ representation (top: CT-ORG dataset, bottom: AbdomenCT-1k dataset). **A**, **C** Sample space from MCIA (see Fig. 2) highlighting the projection of the three individuals with the smallest (blue lines) and largest (red lines) summed distances. The summed distances per individual were calculated from the euclidean distances of each sample to the common center point. **B**, **D** Histograms displaying the distribution of the summed distances per individual. While sample points from some individuals are in close proximity, others are more widely spread

Furthermore, organs from some individuals are generally located in near proximity to each other, whereas organs from others are more widely spread. To quantify this, the distances from each organ to the common center point were summed up. Three individuals each with generally small and high distances are highlighted in Fig. 3A, C. Explicitly, in the CT-ORG dataset the individuals no. 9, 14 and 21 contain the shortest overall distances, whereas the individuals no. 6, 18 and 30 contain the largest overall distances. Likewise, in the AbdomenCT-1k dataset the individuals no. 4, 10, 31 as well as 22, 23, 26 make up for the shortest and largest distances, respectively. The spread may also be solely due to a single organ distancing itself from the center point and other organs, as can be seen for individual no. 22 from the AbdomenCT-1k dataset. The distribution of the summed distance from each organ to their respective center point per individual is presented in Fig. 3B, D. It can be seen that most individuals share the same distance of approximately 1.5 - 3 length units while a small number of individuals cover a shorter or larger distance.

**Fig. 4:**
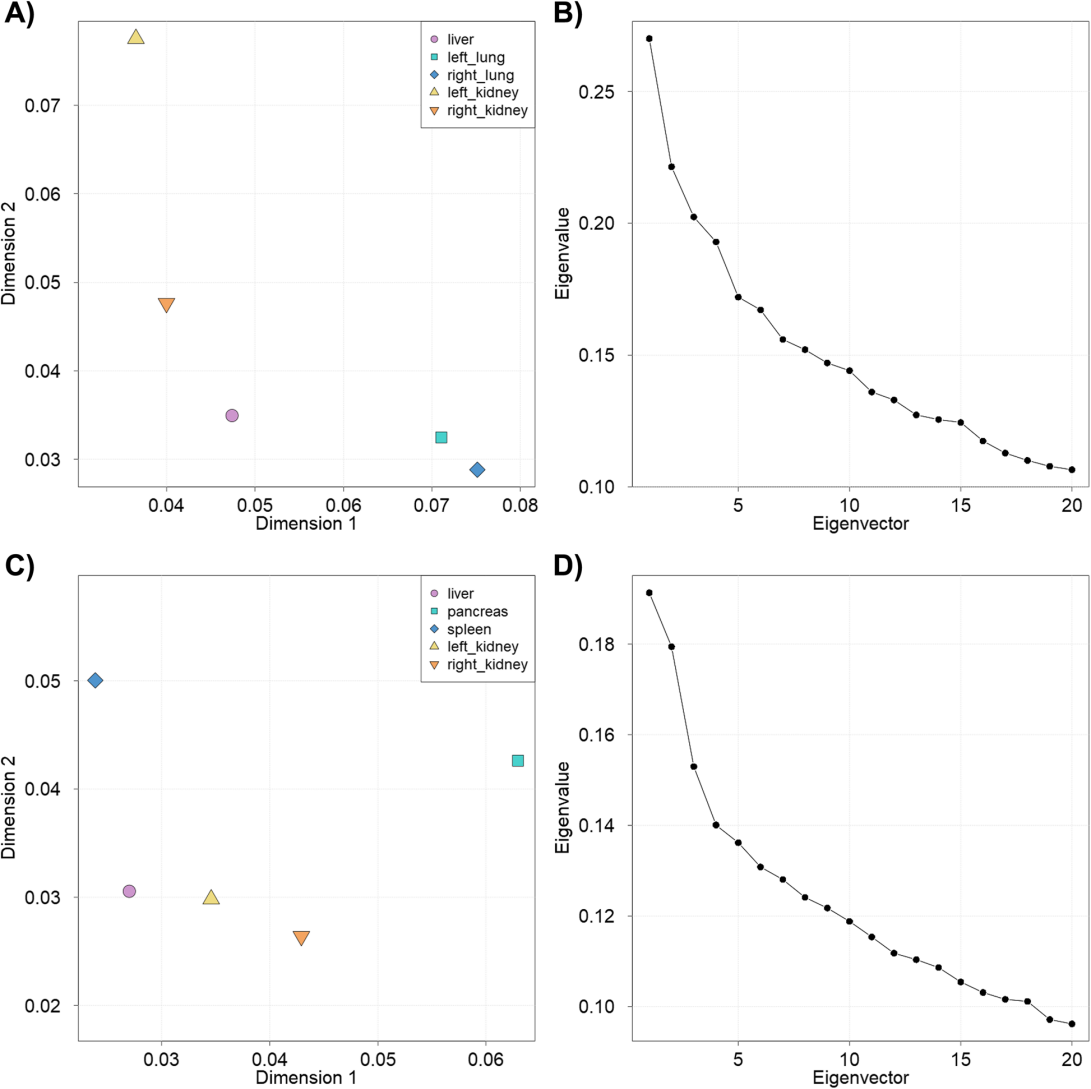
Weighting space and scree plots (top: CT-ORG dataset, bottom: AbdomenCT-1k dataset) from MCIA (see Fig. 2). **A**, **C** The weighting space shows the pseudo-eigenvalues of all feature matrices, demonstrating the amount of variance a feature matrix contributes to each axis. **B**, **D** The scree plot shows the total proportion of variance explained by each axis, sorted in descending order

The amount of variance each feature matrix contributes to a given axis as well as the total amount of variance explained by each axis are presented in Fig. 4. In the CT-ORG dataset, the feature matrices from the left and the right lung contribute mostly to the first axis, whereas the feature matrix from the left kidney contributes the highest to the second axis (Fig. 4 A). In the AbdomenCT-1k dataset, the feature matrix from the pancreas and the spleen contribute mostly to the first and second axis, respectively (Fig. 4C). While the first two axes contain the most variance, the scree plots indicate that further meaning may be revealed exploring additional axes in both datasets (Fig. 4B, D).

### Bagplots, outlier detection and robustness of location

**Fig. 5:**
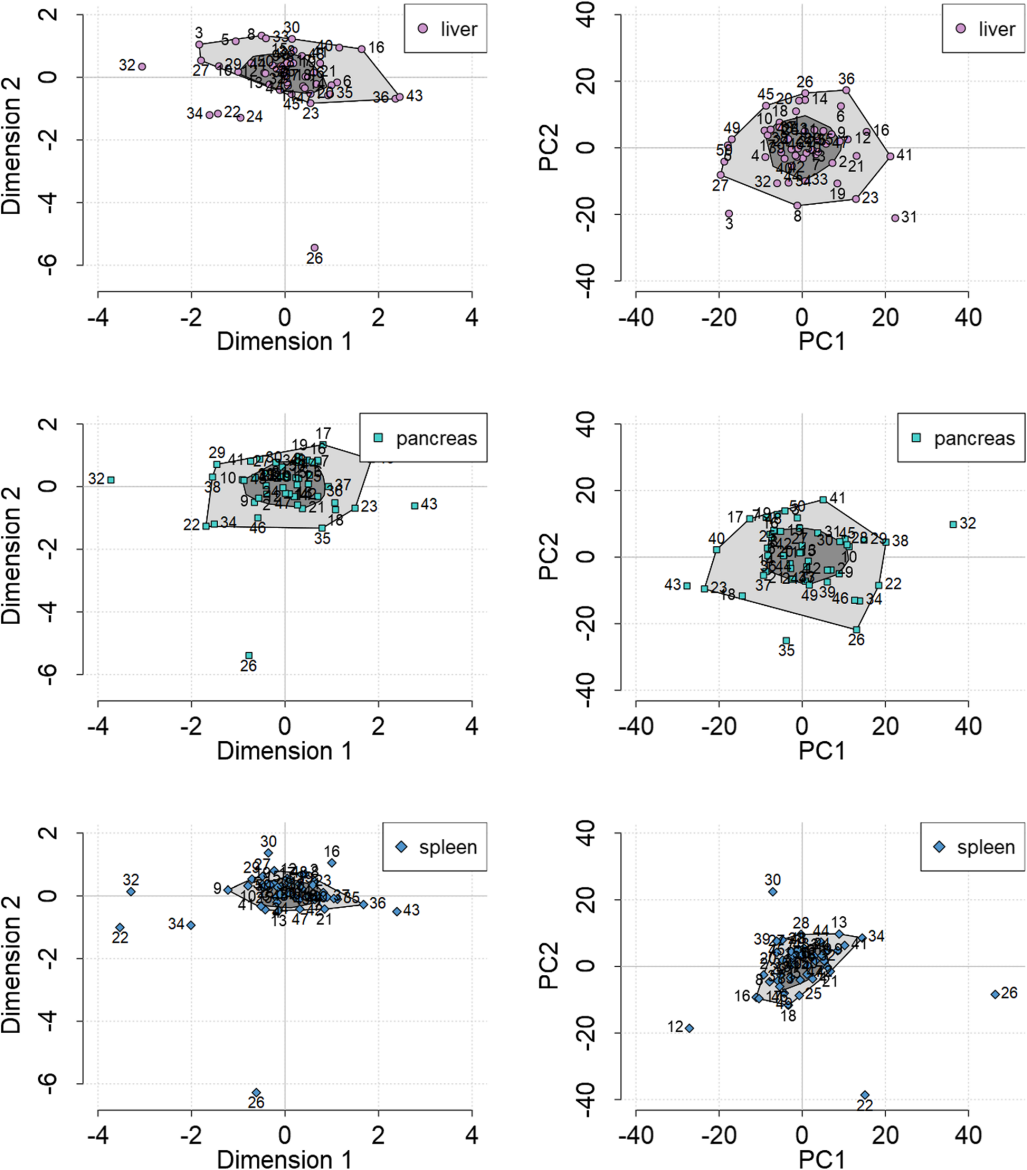
Bagplots applied to the separate 2-dimensional projection (left: MCIA, right: PCA) of feature matrices describing the geometry of multiple organs from the same individuals. Here, a section is shown for the liver, pancreas and spleen from the AbdomenCT-1k dataset. A data point is either located inside the ‘bag’ region (dark grey), inside the ‘fence’ region (light grey) or an outlier (area that is not enclosed). Each individual is labeled by a number. The full images for both datasets, displaying the projection of all organs and comparing all three dimension reduction methods, MCIA, PCA and *t*-SNE, are provided in Supplementary Figs. 1 and 2

**Fig. 6:**
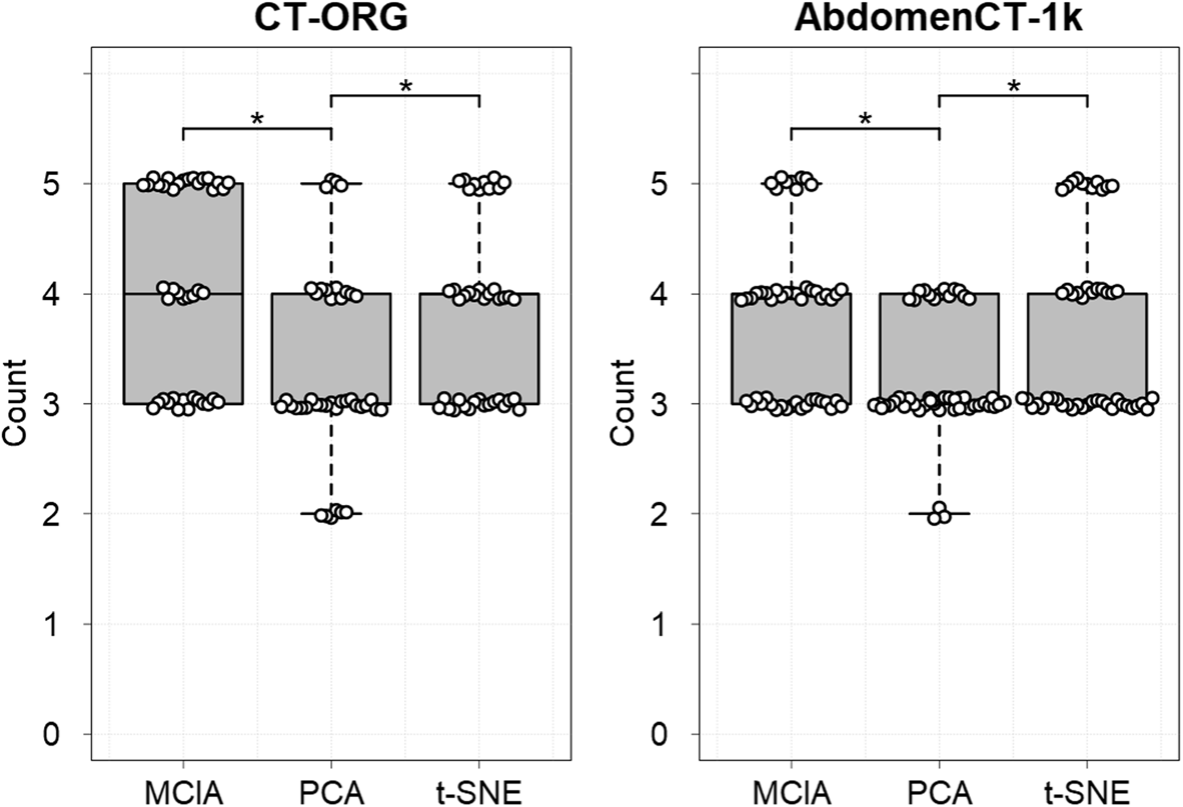
Maximum number of feature matrices (organs) per individual located inside the same 2-dimensional bagplot region, comparing the dimension reduction methods MCIA, PCA and *t*-SNE. For both datasets, CT-ORG (*n* = 41) and AbdomenCT-1k (*n* = 50), MCIA and *t*-SNE show a significantly (*p* < 0.05) higher robustness of multiple organs per individual being projected into the same bagplot region in contrast to PCA. The data points were jittered for visualization purposes: the real count value of a data point corresponds to the nearest integer

Next, the location of individuals within a bagplot was illustrated for each organ, separately, while at the same time contrasting MCIA to other dimension reduction techniques PCA and *t*-SNE (Fig. 5, Suppl. Figs. 2 & 3). At times, the same individuals are shown as outliers both via MCIA and PCA (e.g. individual no. ‘32’ for the pancreas and individual no. ‘22’, ‘26’ and ‘30’ for the spleen, Fig. 5). However, this applies not for all individuals (e.g. individual no. ‘3’ and ‘31’ for the liver, which are detected as outliers in the representation via PCA but not MCIA, Fig. 5). All projections via *t*-SNE from both datasets appear scattered with no outliers present (Suppl. Figs. 2 and 3). Furthermore, it can be seen that the spleen from most individuals (AbdomenCT-1k dataset) are grouped more densely together in comparison to other organs both via MCIA and PCA (Fig. 5). The number of organs per individual being assigned to the same region within a bagplot (‘bag’, ‘fence’ or ‘outlier’ region) were then counted. Figure 6 shows that for both datasets significantly more organs per individual were located within the same region for MCIA and *t*-SNE compared to PCA. This process was repeated with slightly coarser and denser meshes with 500 and 2,000 vertices each. We did not find major changes, with MCIA still being the method with the highest location robustness (Suppl. Fig. 4).

## Discussion

With the help of MCIA, multiple datasets characterising the shape of different organs belonging to the same individuals can be projected into the same low-dimensional space. By doing so, individual outliers within a population can be easily found. At the same time, interrelationships between different organs from one individual can be visualised. This can help to identify abnormal geometry within particular organs of an individual, where the distance to others is unexpectedly large. To better understand which shape descriptors explain the occurrence of an outlier, one may consider the variable space to facilitate interpretation. This information may help to assess whether an outlier is of technical or biological nature.

A technical outlier may arise from errors caused by the applied segmentation method. Manual segmentation is time-consuming and subjective [22, 39]. Therefore, a reliable segmentation algorithm is desirable that may help with computer-assisted visualisation, diagnosis and medical decisions [22, 40]. However, the reproducibility of segmentation algorithms is challenged by the diverse quality of CT scans due to distinct imaging conditions, e.g. from differing technical setups in medicinal centers [22, 41, 42]. In addition, (unseen) diseases may further compromise the generalizbility of segmentation algorithms [22, 43]. Also, low contrast soft tissues, organs with large inter-subject variation and organs with complex morphological structures aggravate the procedure [22, 44]. Many already existing segmentation algorithms are restricted to one particular organ and cannot be applied universally [21, 44, 45]. Most datasets that contain multiple labeled organs have a small sample size, and models utilizing such training data are prone to overfitting [21]. While the automatic segmentation of some organs, such as liver, has been reported to deliver sufficient results, others, such as pancreas, appear more difficult to segment accurately [22, 45]. This may also explain why, in this work, the feature matrix describing the shape of the pancreas contributed the most to the variance of the first axis via MCIA. Also, according to the variable space less curved forms of the pancreas were distinguished from more curved forms. In summary, understanding the nature of an outlier is important to decide on how to deal with them [43]. For example, Xu and colleagues showed that by manually detecting outliers in medical imaging data their abdominal segmentation algorithm may be improved by augmenting the outlier data and adding them to the training set [46]. Ultimately, the premature detection and handling of outliers may benefit the quality of training data and thus the segmentation algorithm itself.

Another technical error may stem from the data processing pipeline, especially the point-set registration. Various algorithms exist: rigid transformation performs translation, rotation and scaling while non-rigid transformation further includes anisotropic scaling and skews to fit one shape into another [47]. The purpose of such algorithms is to find correspondences between point-sets belonging to the same shape family [47, 48]. A statistical shape model (SSM) can then be built from a shape family, describing an average shape together with its variation in shape [48, 49]. A well built SSM can also be utilized as a basis for segmentation [50] or to detect pathologies [51]. The task of point-set registration is especially challenging when facing data noise and deformation [47, 52]. In this work, only the outer boundary from the organs was captured to simplify the registration process. Also, it is noteworthy that there was only a single timepoint per patient recorded. Repeated exposure to CT radiation is harmful to patients and wastes medical resources [53]. Albeit, the organs are flexible and may show various deformations at different timepoints, e.g. due to respiratory motion [54]. For this reason, patients are often given clear instructions for proper breathing technique during image acquisition. Here, no assumption about the state of breathing can be made as the publicly available CT data was collected from various hospitals and locations. Generally, if one intends to analyze the shapes of such organs it is advisable to ensure that images are taken under comparable conditions. On the other hand, our approach could explicitly be used to identify extreme data points that might indicate improper recordings. In addition, features such as the mean curvature may be affected by small distortions from irregularities in the registration process and smoothing algorithms. Ideally, the features derived from the shapes should be directly tied to known phenotypes (e.g. pathologies), for which an experienced radiologist might be consulted. Computing and selecting features that are clinically relevant and more robustly describe the geometry of the shapes as well as including a larger and well defined sample cohort may improve the outcome and interpretation of the representation via MCIA.

More sophisticated approaches to model single- and multi-organ systems exist, that may take into account spatial, functional or physiological inter-organ relationships. For example, they may include information on spatial constraints or biomechanical behaviour of different tissues [1, 55]. The reliability of such models also heavily relies on the quality of preceding segmentation and, where relevant, point correspondences [1, 56]. The here presented approach to illustrate several feature matrices in the same 2-dimensional space and to detect outliers does not depend on overlapping features. Thus, this method may help to easily tackle basic technical issues in advance, e.g. before a segmentation model is trained or a complex multi-organ system is built.

Biological outliers are of interest for diagnostics since they may contain valuable information about the patient [43]. However, as there are no patient data available (e.g. age, sex, health status), no biological interpretation can be made. Besides, in medical contexts incorrect predictions may be especially severe. When the distribution of the training and test data are disparate, the performance of predictive models may significantly decrease [57]. One approach to identify such “out-of-distribution samples” [57] is the combination of dimension reduction with bagplots. On the other hand, as the shapes of abdominal organs may be quite heterogenous among the population [46], one needs to be particularly cautious before dismissing a sample as an outlier. However, in theory, with appropriate knowledge available, biological differences within a predefined population may be quickly identified and highlighted with the here presented method.

## Conclusions

In contrast to univariate measurements such as laboratory values, the specification of reference values or outlier detection is more difficult in multivariate or 3-D data, though both are important for medical decision-making. For cases where multiple datasets per object or individual are available, we have shown that MCIA combined with bagplots is a helpful tool to judge the location of objects or individuals with respect to the data of the whole sample. Yet other dimension reduction methods are helpful to judge the location of individual entities, e.g. organs, as in our data examples.

### Supplementary Information

**Supplementary material 1.**

## Data Availability

The dataset ‘CT-ORG’ analysed during the current study is available in The Cancer Imaging Archive (https://doi.org/10.7937/tcia.2019.tt7f4v7o, [24]). The dataset ‘AbdomenCT-1k’ analysed during the current study is available in the Github repository (https://github.com/JunMa11/AbdomenCT-1K, [22]).

## Abbreviations

CT: Computed tomography
MCIA: Multiple co-inertia analysis
PCA: Principal component analysis
SSM: Statistical shape model
