# Supplementary figures and images for: Dimension reduction and outlier detection of 3-D shapes derived from multi-organ CT images
Source: BMC Med Inform Decis Mak. 2024 Feb 14;24:49. doi: 10.1186/s12911-024-02457-8 (PMC10865689; doi:10.1186/s12911-024-02457-8)

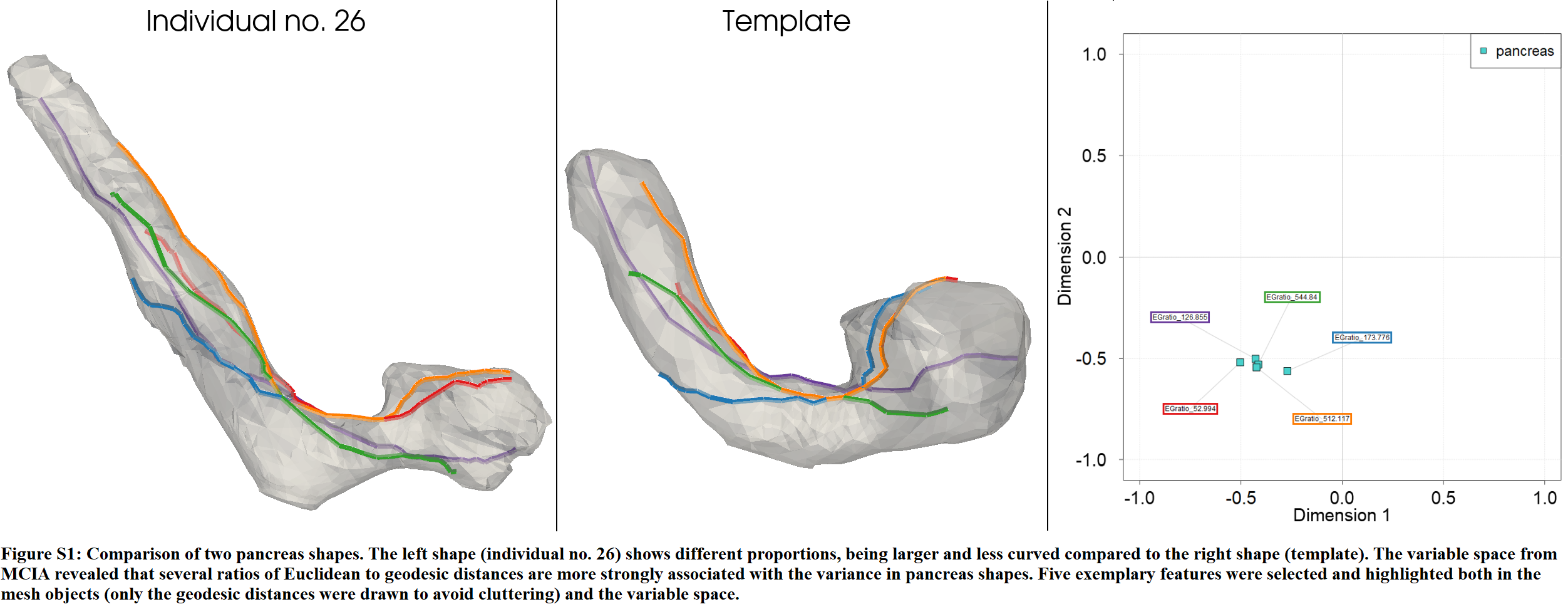

Supplement: Supplementary file 1 — Supplementary material 1. [file 12911_2024_2457_MOESM1_ESM.zip › 12911_2024_2457_MOESM1_ESM/Fig_S1_ESM.png]

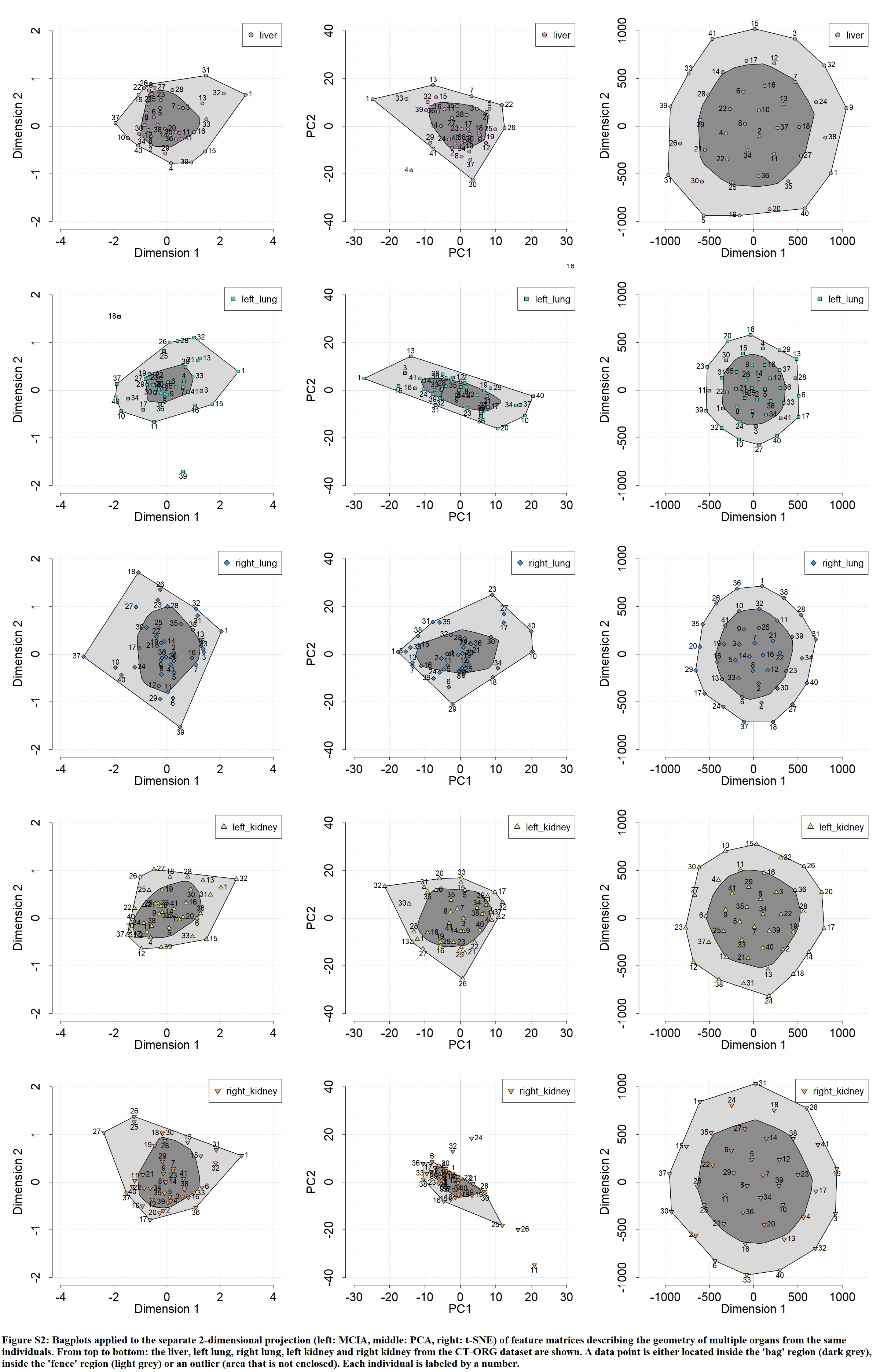

Supplement: Supplementary file 1 — Supplementary material 1. [file 12911_2024_2457_MOESM1_ESM.zip › 12911_2024_2457_MOESM1_ESM/Fig_S2_ESM.png]

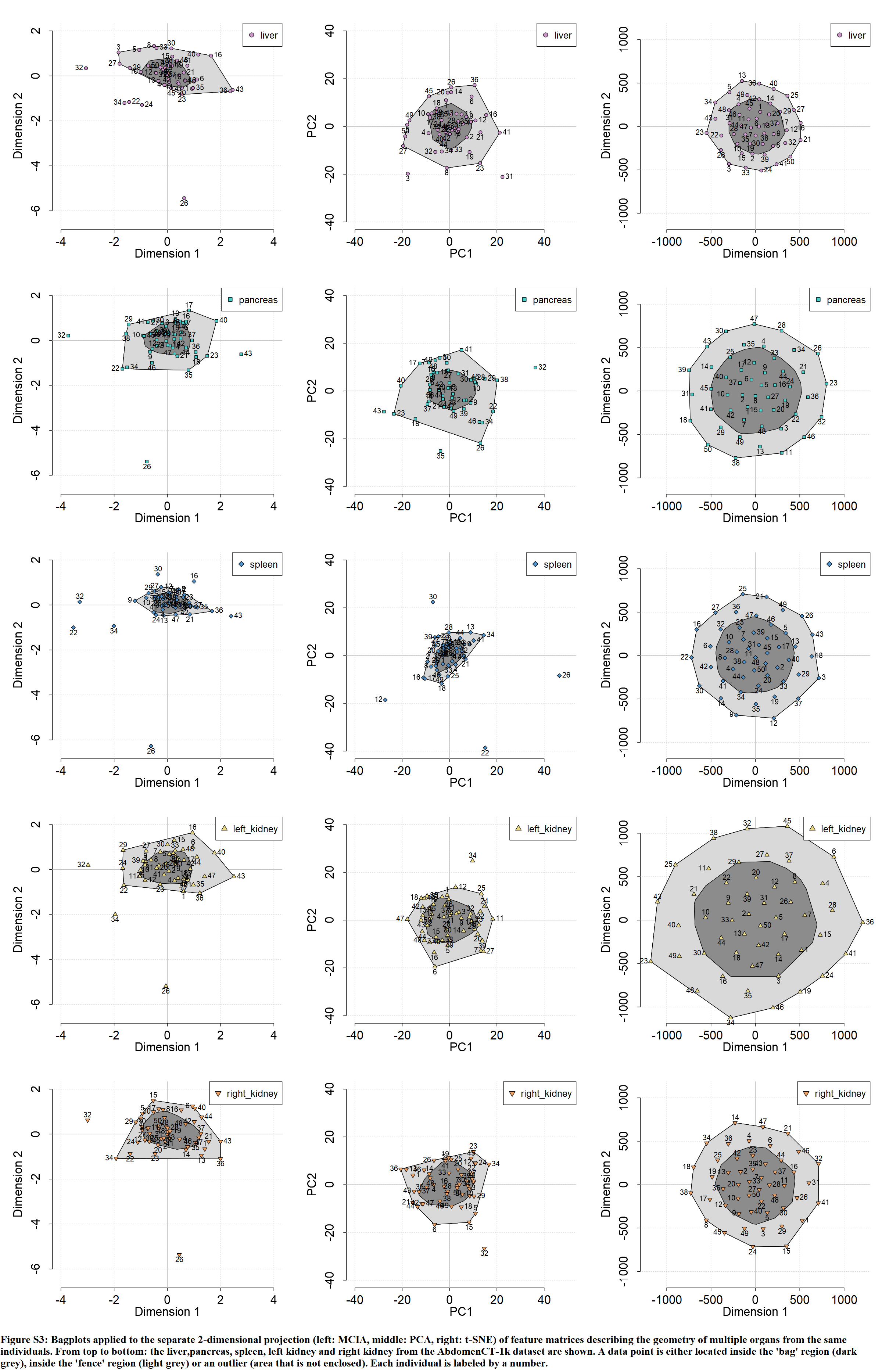

Supplement: Supplementary file 1 — Supplementary material 1. [file 12911_2024_2457_MOESM1_ESM.zip › 12911_2024_2457_MOESM1_ESM/Fig_S3_ESM.png]

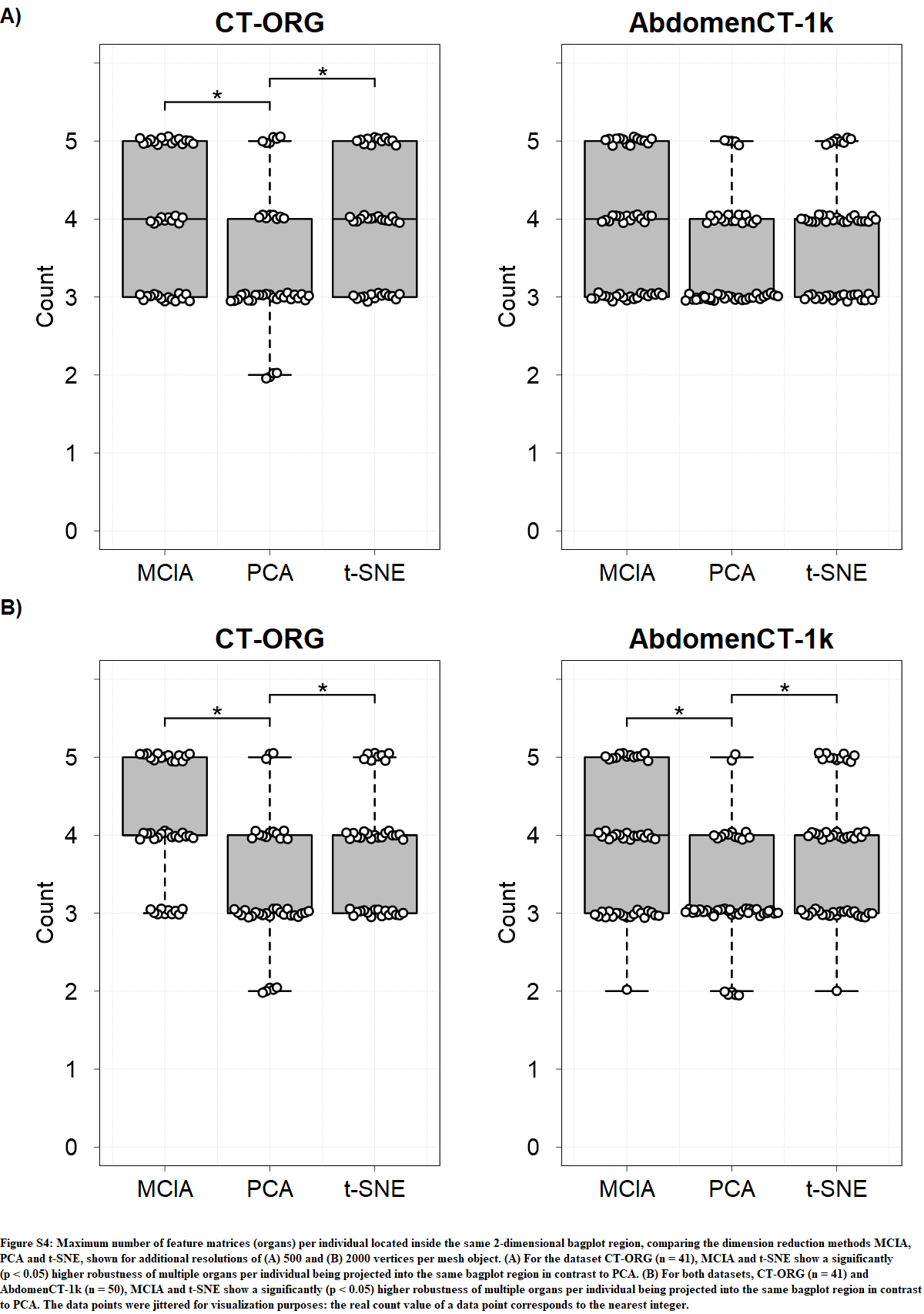

Supplement: Supplementary file 1 — Supplementary material 1. [file 12911_2024_2457_MOESM1_ESM.zip › 12911_2024_2457_MOESM1_ESM/Fig_S4_ESM.png]
